# Kakkonto-Induced Pneumonitis: A Case Report

**DOI:** 10.7759/cureus.110627

**Published:** 2026-06-10

**Authors:** Ikuko Ooka, Tetsu Hirakawa, Yoshito Temma, Tetsuji Yamaguchi, Tomokazu Ishitobi

**Affiliations:** 1 Department of Internal Medicine, Yoshida General Hospital, Akitakata, JPN; 2 Department of Molecular and Internal Medicine, Graduate School of Biomedical and Health Sciences, Hiroshima University, Hiroshima, JPN

**Keywords:** corticosteroids, drug-induced pneumonitis, drug lymphocyte stimulation test, japanese herbal medicine, kakkonto

## Abstract

A 76-year-old woman with a history of polymyalgia rheumatica, dyslipidemia, and osteoporosis was admitted due to a one-week history of productive cough and was initially diagnosed with bacterial pneumonia. Despite antibiotic therapy, her clinical condition worsened, leading to respiratory failure. Further history-taking revealed that she had been taking kakkonto, a Japanese herbal medicine (JHM), for the preceding 4.5 months as a self-initiated measure to prevent infection. The patient was eventually diagnosed with kakkonto-induced pneumonitis. Kakkonto was discontinued, and systemic corticosteroid therapy was initiated, resulting in rapid improvement. The peripheral blood drug-induced lymphocyte stimulation test was positive for kakkonto. Because the clinical and radiological findings of JHM-induced pneumonitis are nonspecific, careful medication history-taking, particularly for JHM, is essential for diagnosing unexplained pneumonitis.

## Introduction

Drug-induced pneumonitis occurs following exposure to numerous agents, including antineoplastic drugs (e.g., gefitinib and immune checkpoint inhibitors), immunomodulators (e.g., methotrexate), antiarrhythmics (e.g., amiodarone), and antibiotics (e.g., nitrofurantoin). Its clinical manifestations vary widely, ranging from mild respiratory symptoms to rapidly progressive respiratory failure and death [1]. In most cases, no clinical, laboratory, radiological, or pathological findings are pathognomonic for this condition. Kakkonto, a Japanese herbal medicine (JHM) consisting of seven crude drugs, is widely used in Japan for early-stage common cold symptoms, shoulder stiffness, and neuralgia [2]. Although JHMs are considered relatively safe with few adverse effects, reports of JHM-induced pneumonitis have been increasing [3,4].

Herein, we report a case of kakkonto-induced pneumonitis in which careful history-taking was pivotal to establishing the diagnosis. To date, four cases of kakkonto-induced pneumonitis have been reported, all of which developed within one month after the initiation of kakkonto. In contrast, the present case developed after 4.5 months of continuous kakkonto use. This case suggests that kakkonto-induced pneumonitis can occur even after several months of use and highlights the importance of awareness of drug-induced pneumonitis and careful medication history-taking for an accurate diagnosis.

## Case presentation

A 76-year-old woman presented with a one-week history of productive cough and lower chest pain upon coughing. Her medical history included polymyalgia rheumatica diagnosed approximately nine months before admission, dyslipidemia of unknown duration, and osteoporosis diagnosed approximately six months before admission. She had been taking celecoxib for approximately eight months and eldecalcitol for approximately six months before admission. She had never smoked and had no history of pulmonary disease or allergies. On presentation, her vital signs were as follows: blood pressure of 107/69 mmHg, pulse rate of 99 beats/min, body temperature of 36.2 °C, respiratory rate of 20 breaths/min, and oxygen saturation of 95% on room air. Auscultation revealed coarse crackles predominantly in the left lower lung field. Physical examination revealed no findings suggestive of heart failure or connective tissue disease. Laboratory tests showed a white blood cell count of 10,900/μL and a C-reactive protein level of 20.52 mg/dL (Table 1).

**Table 1:** Laboratory findings at admission Alb, albumin; ALP, alkaline phosphatase; ALT, alanine aminotransferase; AST, aspartate aminotransferase; Bas, basophils; BUN, blood urea nitrogen; CK, creatine kinase; Cl, chloride; Cre, creatinine; CRP, C-reactive protein; Eo, eosinophils; Glu, glucose; γ-GTP, gamma-glutamyl transpeptidase; Hb, hemoglobin; HbA1c, hemoglobin A1c; LDH, lactate dehydrogenase; Lym, lymphocytes; Mon, monocytes; Neu, neutrophils; PLT, platelet; RBC, red blood cell; T-Bil, total bilirubin; TP, total protein; WBC, white blood cell

Parameter	Result	Unit	Reference range
Hematology			
WBC	10,900	/μL	4,000-9,000
Neu	81.2	%	40-70
Lym	8.4	%	20-45
Mon	8.3	%	2-10
Eo	1.9	%	1-6
Bas	0.2	%	0-1.2
RBC	374	×10^4^/μL	380-580
Hb	11.5	g/dL	12-15
PLT	48.0	×10^4^/μL	16.4-35.4
Chemistry			
TP	6.7	g/dL	6.6-8.1
Alb	2.9	g/dL	4.1-5.1
BUN	11.3	mg/dL	8-20
Cre	0.51	mg/dL	0.46-79
Na	132.7	mEq/L	138-145
K	4.14	mEq/L	3.6-4.8
Cl	93.4	mEq/L	101-108
T-Bil	0.59	mg/dL	0.4-1.5
AST	20	U/L	13-30
ALT	15	U/L	7-23
LDH	168	U/L	124-222
ALP	159	U/L	38-113
γ-GTP	70	U/L	9-32
CK	46	U/L	41-153
Glu	106	mg/dL	73-109
HbA1c	6.1	%	4.9-6.0
CRP	20.52	mg/dL	0.00-0.14

Chest radiography demonstrated consolidations predominantly in the left upper lung field with a smaller infiltrative opacity in the right upper lung field (Figure 1).

**Figure 1:**
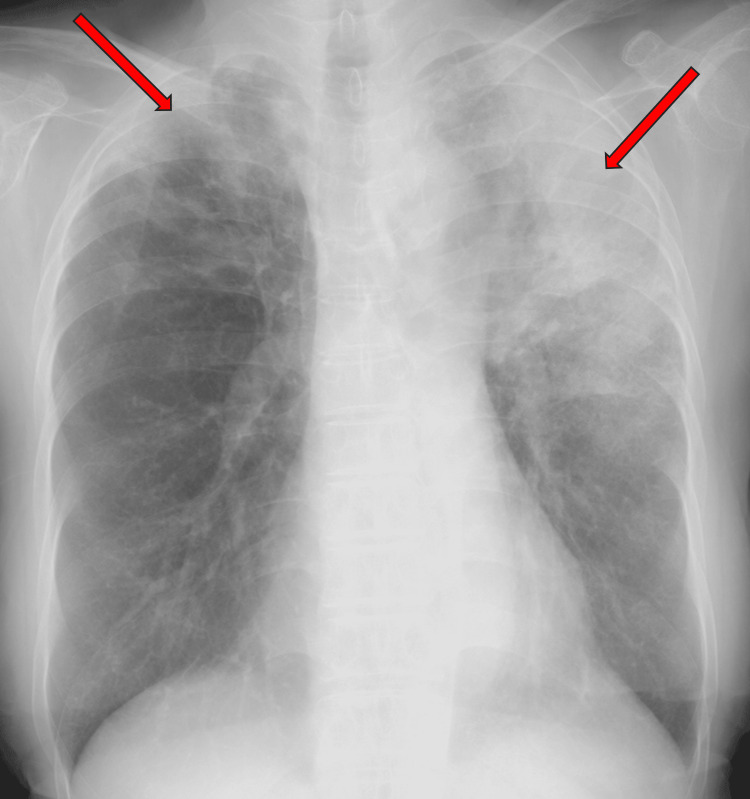
The chest radiograph demonstrated consolidations predominantly in the left upper lung field, with a smaller infiltrative opacity in the right upper lung field (red arrows)

Chest computed tomography (CT) demonstrated pulmonary consolidations and ground-glass opacities in both lungs, predominantly in the left lung, accompanied by a small left pleural effusion (Figure 2).

**Figure 2:**
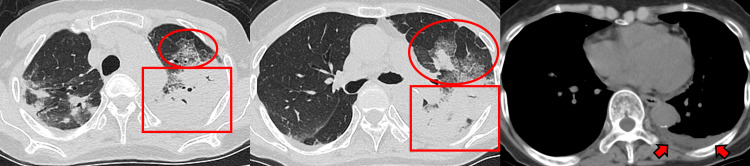
Chest CT on admission showing pulmonary consolidations (boxes) and ground-glass opacities (circles) in both lungs, predominantly in the left lung, with a small left pleural effusion (red arrows) CT, chest computed tomography

Additional tests, including Krebs von den Lungen-6, autoantibodies associated with connective tissue diseases, tumor markers, β-D-glucan, and the T-SPOT test, were all unremarkable (Table 2).

**Table 2:** Additional laboratory findings Ab, antibody; Ag, antigen; ANA, antinuclear antibody; CA19-9, carbohydrate antigen 19-9; CCP, cyclic citrullinated peptide; CEA, carcinoembryonic antigen; IgE, immunoglobulin E; KL-6, Krebs von den Lungen-6; MMP-3, matrix metalloproteinase-3; ProGRP, pro-gastrin-releasing peptide; RF, rheumatoid factor

Parameter	Result	Unit	Reference range
Serological tests			
KL-6	194	U/mL	0-499.9
IgE	139	mg/dL	0-170
ANA	< 1:40		< 1:40
RF	2.7	U/mL	0-15
Anti-CCP Ab	3.4	U/mL	0.5-4.4
PR3-ANCA	< 0.6	U/mL	Negative
MPO-ANCA	< 0.2	U/mL	0.00-3.499
MMP-3	29.6	ng/mL	17.3-59.7
Tumor markers			
CEA	<1.7	ng/mL	0-5
CA19-9	10	U/mL	0-37
ProGRP	27.3	pg/mL	0-80.99
Microbiological tests			
β-D-glucan	17.5	pg/mL	0-20
T-SPOT	Negative		Negative
Influenza A Ag	Negative		Negative
Influenza B Ag	Negative		Negative
SARS-CoV-2 Ag	Negative		Negative
Pneumococcal urinary Ag	Negative		Negative
*Legionella *urinary Ag	Negative		Negative
Blood culture	Negative		Negative
Sputum culture	*Klebsiella pneumoniae*, *Acinetobacter baumannii*		

Based on these findings, bacterial pneumonia was initially suspected, and intravenous ampicillin/sulbactam (3 g every eight hours) was administered for five days, along with oral azithromycin (500 mg once daily) for three days. Following the sputum culture results (Table 2), antimicrobial therapy was subsequently escalated to intravenous piperacillin/tazobactam (4.5 g every six hours) for five days.

However, inflammatory markers and radiological findings progressively worsened, leading to respiratory failure requiring oxygen supplementation at 2 L/min via nasal cannula. Follow-up chest CT revealed worsening pulmonary consolidations and an increase in pleural effusion (Figure 3), although pleural fluid examination could not be performed because the amount of pleural effusion was insufficient.

**Figure 3:**
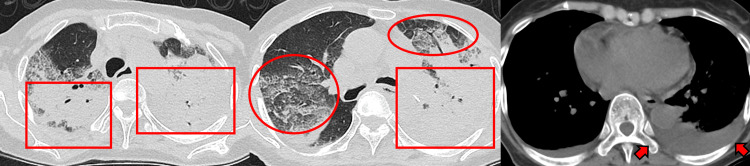
Chest CT on hospital day seven showing progression of pulmonary consolidations (boxes) and ground-glass opacities (circles) with an increased left pleural effusion (red arrows) CT, chest computed tomography

Further history taking revealed that the patient had been taking kakkonto continuously for the preceding approximately 4.5 months as a self-initiated measure to prevent infection. Given the suspicion of drug-induced pneumonitis and the progressive worsening of the patient's respiratory status and radiological findings, kakkonto was discontinued, and systemic corticosteroid therapy was initiated with intravenous methylprednisolone (1,000 mg/day for three days), followed by oral prednisolone (30 mg/day). Bronchoscopy was not performed because of the rapidly worsening respiratory status. Oxygen therapy was discontinued after six days of the initiation of corticosteroid therapy, and radiological findings gradually improved in parallel with the clinical course. Drug-induced lymphocyte stimulation test (DLST) using peripheral blood was positive for kakkonto (stimulation index: 234%; reference range: < 180%), supporting the diagnosis of kakkonto-induced pneumonitis. The clinical course is summarized in Figure 4.

**Figure 4:**
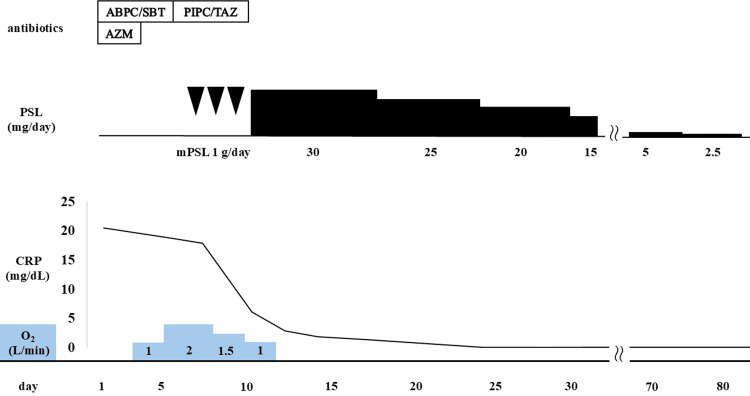
Clinical course of the patient ABPC/SBT, ampicillin/sulbactam; AZM, azithromycin; CRP, C-reactive protein; mPSL, methylprednisolone; O_2_, oxygen; PIPC/TAZ, piperacillin/tazobactam; PSL, prednisolone

Corticosteroids were gradually tapered over 11 weeks and then discontinued after resolution of pulmonary consolidations and pleural effusion on chest CT (Figure 5). After completion of corticosteroid therapy, no recurrence of pneumonitis was observed.

**Figure 5:**
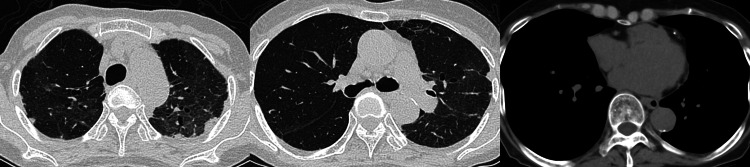
Chest CT after treatment showing resolution of pulmonary consolidations and ground-glass opacities with disappearance of the left pleural effusion CT, Chest computed tomography

## Discussion

Kakkonto has been reported to have antiviral and anti-inflammatory properties and is one of the most commonly used JHMs in Japan for early-stage common cold symptoms, shoulder stiffness, and neuralgia [5,6]. Although JHMs have long been considered to cause few adverse effects, reports of JHM-induced pneumonitis have increased in recent years [3,4]. Because JHMs are composed of multiple crude drugs, identifying the causative ingredient is often difficult; however, several reported cases of JHM-induced pneumonitis involve formulations containing ougon or kanzo, which may be key contributors to pneumonitis [3,7]. Kakkonto consists of seven crude drugs (kakkon, maou, keihi, syakuyaku, syoukyou, taisou, and kanzo), and kanzo has been implicated as a potential contributor to kakkonto-induced pneumonitis. Generally, the pathogenesis of drug-induced pneumonitis involves both direct cytotoxic injury and allergic mechanisms [8,9]. Although the precise mechanisms of JHM-induced pneumonitis remain unclear, they are generally considered to be mediated predominantly by allergic reactions (type I, III, and IV hypersensitivity according to the Coombs and Gell classification) rather than by direct toxic injury [4,10].

Although most cases of JHM-induced pneumonitis have been reported to occur within three months of initiating JHM, pneumonitis may also develop after long-term use beyond three months, as in the present case [3,11]. In addition, pharmacovigilance data suggest a dose-dependent association between drug-induced pneumonitis and intake of certain crude drugs [12]. Therefore, drug-induced pneumonitis should always be considered in patients taking JHMs, particularly those with long-term use.

Diagnosing JHM-induced pneumonitis is challenging because its clinical manifestations and radiological findings are nonspecific and require differentiation from infection and other inflammatory lung diseases, as observed in the present case. Fever, cough, and dyspnea are common symptoms of JHM-induced pneumonitis, and bilateral ground-glass opacities are frequently observed on chest CT [3]. To date, four cases of kakkonto-induced pneumonitis have been reported (Table 3), and both symptoms (e.g., cough and dyspnea) and radiological findings (e.g., diffuse ground-glass opacities and consolidation) were largely nonspecific [13-16].

**Table 3:** Reported cases of kakkonto-induced pneumonitis BAL, bronchoalveolar lavage; BALF, bronchoalveolar lavage fluid; BAT, basophil activation test; DLST, drug-induced lymphocyte stimulation test; F, female; M, male; mPSL, methylprednisolone; PSL, prednisolone; TBLB, transbronchial lung biopsy

Reference	Sex	Age	Duration of kakkonto use before onset	Symptoms	Radiologic findings	Additional diagnostic tests	Treatment
[13]	M	53	2 weeks	Dry cough and dyspnea on exertion	Panlobular ground-glass opacities	BAL, blood DLST (+)	Corticosteroid (mPSL 250 mg)
[14]	F	63	3 weeks	Dry cough and dyspnea on exertion	Bilateral patchy consolidations and ground-glass opacities along the bronchovascular bundles	Blood DLST (+), BAT (+)	Corticosteroid (mPSL 1000 mg)
[15]	F	74	1 month	Dyspnea on exertion	Diffuse ground-glass opacities and consolidations	BAL, TBLB, blood DLST (−), BALF DLST (+)	Corticosteroid (PSL 40 mg)
[16]	F	79	The day after inhalation	Fever and dyspnea	Bilateral diffuse ground-glass opacities with an airway distributed mosaic pattern	BAL, blood DLST (+)	Discontinuation of kakkonto alone
This case	F	76	4.5 months	Productive cough and lower chest pain	Bilateral consolidations, ground-glass opacities, and pleural effusion	Blood DLST (+)	Corticosteroid (mPSL 1000 mg)

In the present case, pleural effusion was also observed. Although pleural fluid examination could not be performed because the amount of pleural effusion was insufficient, the effusion resolved promptly after discontinuation of kakkonto and initiation of corticosteroid therapy. A previous study reported that pleural effusion on CT was observed in 11% of patients with JHM-induced pneumonitis [3]. These findings suggest that pleural effusion, although uncommon, may occur in some patients with kakkonto-induced pneumonitis.

JHM-induced pneumonitis can be evaluated using several diagnostic tests; however, diagnostic protocols remain controversial. DLST using peripheral blood is considered relatively safe and is commonly used as a supportive test for drug-induced pneumonitis. In the present case, DLST for kakkonto was positive. Nevertheless, the results of the DLST should be interpreted with caution and used only as an adjunct because false-positive results have been reported, particularly with JHMs. JHMs contain various compounds other than the active ingredients and may also contain mitogenic substances, such as lectins and mitogens, which may lead to false-positive results [17]. Recently, alternative approaches, such as DLST using bronchoalveolar lavage fluid (BALF) and basophil activation tests, have been reported [14,15]. Notably, Nishiyama et al. reported a case of kakkonto-induced pneumonitis in which the DLST using peripheral blood was negative, and the DLST using BALF was positive [15]. A drug provocation (rechallenge) test provided strong supportive evidence of causality. However, this is rarely performed because hypersensitivity reactions can be life-threatening, raising safety and ethical concerns. In addition, in the present case, although bronchoscopy was initially planned for further evaluation and to exclude alternative diagnoses, the procedure could not be performed because of rapidly worsening respiratory failure. Despite these limitations, we excluded alternative diagnoses as thoroughly as possible, and the patient showed rapid improvement after discontinuing kakkonto and initiating systemic corticosteroids, with no relapse after steroid withdrawal. Taken together, these findings supported the diagnosis of kakkonto-induced pneumonitis.

Although the prognosis of JHM-induced pneumonitis is generally favorable, the clinical course can be severe in a subset of patients. In a previous report, 36% of patients achieved complete recovery with discontinuation of the causative agent alone, whereas the remaining patients required systemic corticosteroids and/or immunosuppressive therapy [3]. Furthermore, mechanical ventilatory support was needed in 18% of cases, and 4% did not survive. In the present case, the patient developed rapidly progressive respiratory deterioration; however, systemic corticosteroid therapy was promptly initiated, resulting in clinical improvement without progression to severe respiratory failure. Although evidence supporting corticosteroid pulse therapy for drug-induced pneumonitis remains limited, it was empirically selected in the present case to achieve prompt control of pulmonary inflammation, given the progressive worsening of the patient's respiratory status and radiological findings [18]. These findings underscore that delayed recognition may result in progressive pneumonitis and severe outcomes. Therefore, awareness of JHM-induced pneumonitis and prompt discontinuation of suspected JHM are crucial to prevent disease progression and improve prognosis.

## Conclusions

This case report highlights the importance of awareness of drug-induced pneumonitis and careful medication history taking, including the use of JHMs, in patients with unexplained pneumonitis. Because the clinical and radiological manifestations of drug-induced pneumonitis are diverse and nonspecific, diagnosis based on symptoms and imaging alone is often challenging. Kakkonto is one of the most commonly used JHMs; therefore, drug-induced pneumonitis should always be considered in patients who develop pneumonitis while taking kakkonto.
